# Mapping malaria incidence distribution that accounts for environmental factors in Maputo Province - Mozambique

**DOI:** 10.1186/1475-2875-9-79

**Published:** 2010-03-21

**Authors:** Orlando P Zacarias, Mikael Andersson

**Affiliations:** 1 Department of Mathematics and Informatics (DMI), Eduardo Mondlane University and Department of Computer and System Sciences (DSV), Stockholm University, Sweden; 2 Department of Mathematics, Division of Mathematical Statistics, Stockholm University, Sweden

## Abstract

**Background:**

The objective was to study if an association exists between the incidence of malaria and some weather parameters in tropical Maputo province, Mozambique.

**Methods:**

A Bayesian hierarchical model to malaria count data aggregated at district level over a two years period is formulated. This model made it possible to account for spatial area variations. The model was extended to include environmental covariates temperature and rainfall. Study period was then divided into two climate conditions: rainy and dry seasons. The incidences of malaria between the two seasons were compared. Parameter estimation and inference were carried out using MCMC simulation techniques based on Poisson variation. Model comparisons are made using DIC.

**Results:**

For winter season, in 2001 the temperature covariate with estimated value of -8.88 shows no association to malaria incidence. In year 2002, the parameter estimation of the same covariate resulted in 5.498 of positive level of association. In both years rainfall covariate determines no dependency to malaria incidence. Malaria transmission is higher in wet season with both covariates positively related to malaria with posterior means 1.99 and 2.83 in year 2001. For 2002 only temperature is associated to malaria incidence with estimated value 2.23.

**Conclusions:**

The incidence of malaria in year 2001, presents an independent spatial pattern for temperature in summer and for rainfall in winter seasons respectively. In year 2002 temperature determines the spatial pattern of malaria incidence in the region. Temperature influences the model in cases where both covariates are introduced in winter and summer season. Its influence is extended to the summer model with temperature covariate only. It is reasonable to state that with the occurrence of high temperatures, malaria incidence had certainly escalated in this year.

## Background

Malaria is the primary cause of mortality in Mozambique, resulting in an estimated 44,000-67,000 malaria-specific deaths each year for all age groups [1]. Transmission intensity varies from region to region, with high incidence in areas where climatic conditions are favourable towards its development and transmission, whereas some drier parts of the country are epidemic-prone. About six million reported cases of malaria are accounted every year, with 44% of all outpatient consultations and 65% of all paediatric hospital admissions. It is the leading cause of death among children admitted to paediatric services in Mozambique, with figures of 32% in year 1998, 42% in year 1999 and 40% in year 2000. Malaria is also a major problem affecting pregnant women in rural areas (like Maputo province), with parasites infection rates of around 20% and a level of 30% among first pregnancies [1,2]. Health coverage is very low in the country, with 56% of population being over an hour away from the nearest health centre. In rural areas the figure is higher (72%) than in urban areas (14%). On the other hand, the level of *Plasmodium falciparum *resistance to drugs like chloroquine has been reported throughout most of the country [3-5]. Currently health authorities have dropped its use in the country.

A number of studies modelling the spatial distribution of malaria and other tropical diseases in Africa using environmental data have adopted Bayesian approach [6-9]. The flexibility and robustness provided by Bayesian methods has led to the increase of applications on disease mapping, spatial statistics and decision-making [10]. These methods can incorporate spatial correlation and the uncertainty into modelling process is achieved by modelling the observed data and any unknown parameter as random variables. Although Bayesian methods were initially derived for use in small-area analyses of chronic non-infectious disease [10], recently they have been used in the studies of geographical distribution of malaria [11] and other tropical diseases. Geographic modelling of malaria distribution is central for understanding spatial and and/or spatio-temporal patterns that may help to identify discrepancies in disease burden among different districts in Mozambique. These patterns often reflect a composition of human host factors, heterogeneities in vector distribution and human-vector contact [12]. The capability of uncertainty assessment within the Bayesian approach has increased its usage in all aspects of disease mapping. Estimates of uncertainty obtained as model outputs are easily included in the map production in relation to malaria disease control strategy, as to facilitate a strategy based on geographic risk stratification. This may allow for more informed and objective decision making from disease control programme managers. A more detailed knowledge of malaria incidence risk in the region may also serve as a basis for an increase of health service provision and improved targeted malaria control. While spatial analytical methods are regarded as an attractive research objective [9], they have been rarely applied in context of district geographical health analyses in Mozambique, especially as tools for enhanced planning and implementation of disease control programmes.

The aim of this study was to conduct a spatial statistical analysis of malaria incidence to identify important predictor variables and to produce a malaria distribution map of Maputo province, illustrating the variation in malaria risk. It is thus investigated whether there is any association between malaria incidence and environmental variables, temperature and rainfall in the region.

## Methods

### Study area

**Figure 1:**
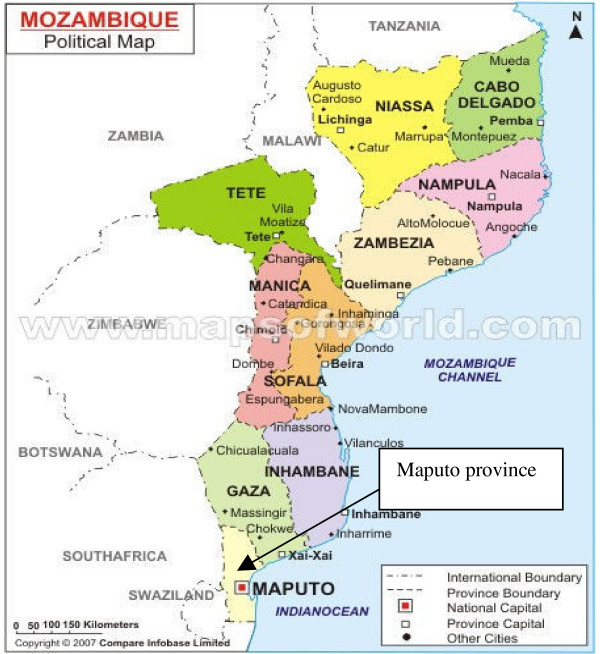
**Mozambique map provided by **[20], **with an indication of study region (Maputo province)**.

Maputo province (study region) has an area of 23,669 km^2^. Its boundaries are Gaza province to the north, South Africa to the south, Swaziland to the west and Indian Ocean to the east. With population size of around 900,000 people, the province exhibits a density of 44 inhabitants per square kilometres [2]. It is sub-divided into eight administrative districts. Figure 1, shows the Mozambique location map highlighting the study area - Maputo province. It is composed of the districts of Magude, Moamba, Manhiça, Marracuene, Matola, Boane, Namaacha and Matutuine.

The regional health network comprises 92 health facilities [2]. Of these, three are rural district hospitals, 23-50 health posts located at small rural administrative areas within the province, which offer very basic health service to their community, and a set of health centres.

### Data

**Figure 2:**
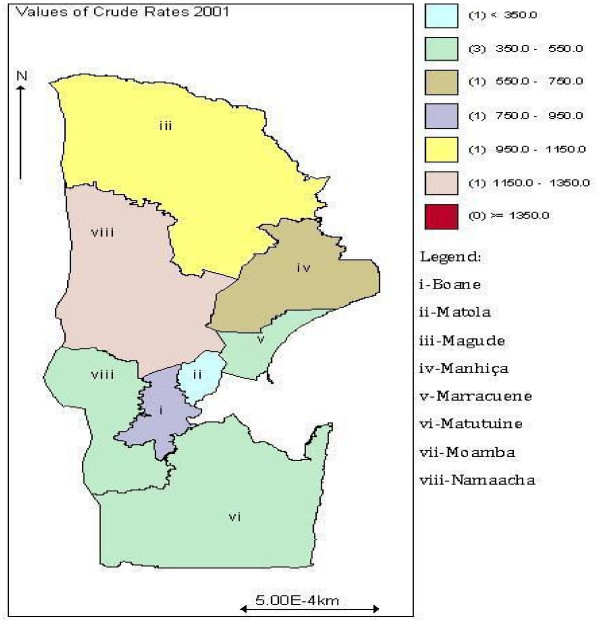
**Crude incidence density of malaria in year 2001**.

**Figure 3:**
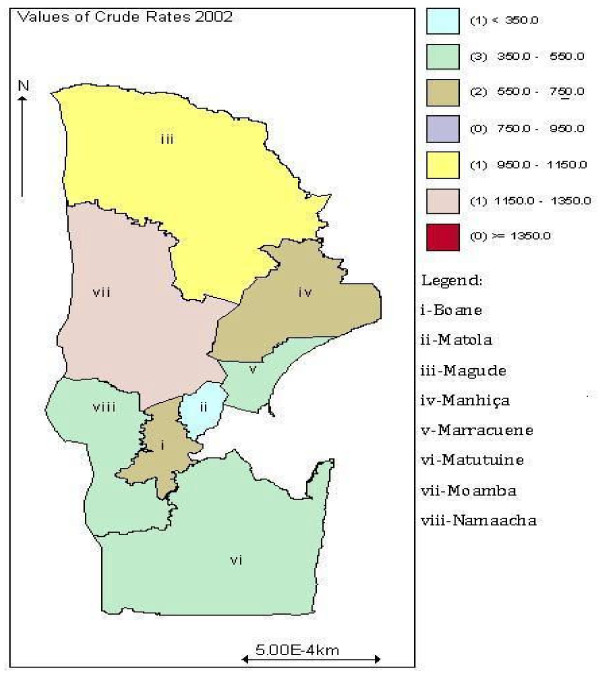
**Crude incidence density of malaria in year 2002**.

Cases of malaria are scrutinized daily in various health centres and rural hospitals and gathered together with other diseases occurrences to produce the BES, and then summarized into annual reports. The cases thus aggregated as counts of disease events are channelled to NMCP where are centralized by district within Maputo province [1]. This study covers data from years 2001 and 2002. They included cases confirmed either by a microscopy or by a rapid (inexpensive) diagnostic test, and also unconfirmed cases with symptoms similar to malaria diagnosed by health-trained personnel (clinical malaria cases). The spatial crude incidence density map of malaria for years 2001 and 2002 in Maputo province is illustrated in Figures 2 and 3 respectively.

**Figure 4:**
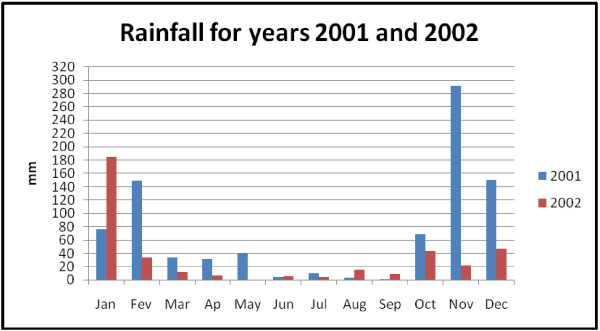
**Monthly rain precipitation in Maputo province**.

**Figure 5:**
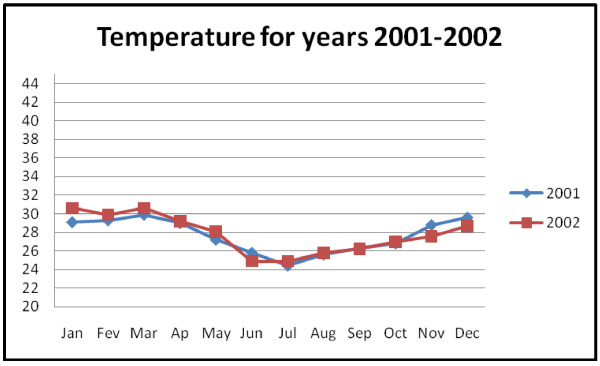
**Average maximum temperature in Maputo province**.

The Mozambique tropical to sub-tropical climate is divided into the dry (winter) and rainy (summer) seasons, with Maputo province being sub-tropical. Summer season goes from October to March, while the winter lasts from April to September. Two years climatic data, monthly averages maximum temperature and rainfall were obtained from INAM and used as covariates. These data are read twice daily at the Benfica meteorological station in Maputo city. They are accumulated per month and average values calculated to yield a monthly weather bulletin. Rainfall in the study region is highly seasonal, with highest values of 290.9 mm and 184.8 mm in November 2001 and January 2002 respectively (Figure 4). Low amounts of rain were reported in months June to September in both years. The air temperature reaches peak values in January-March and December, with low values in June-July in both years (Figure 5). Moreover, it is known that malaria vector does not develop at low temperatures similar to those that occur in some regions of Maputo province in the dry season, due to many reasons including reduction of breeding pools. This may contribute to the reduction of malaria incidence levels. On the other hand, the available malaria data does not capture this seasonality. Thus, the use of stratification is a way to examine the characteristics of the incidence of malaria in each season.

### Modelling

Malaria distribution is governed by a set of factors relating the parasite, the vector and the human host [13]. Environmental and climatic factors are among the most important particularly, temperature, rain precipitation, humidity, vegetation, stationary water pools and human-vector interaction, as they affect the habitat and vector breeding sites. Monthly maximum temperature and rainfall climatic data were used for modelling and mapping malaria incidence risk. To analyse the relationship between environmental factors and malaria cases, a Poisson model in Statistical Package R [14] was fitted. Both covariates showed a significant association with malaria cases with *P *< 0.001. Climatic data is aggregated into seasonal averages and are separately modelled for each year. This resulted in two seasonal dependent models for each year.

Let's sub-divide the geographic domain of Maputo province into *i *= 1,..., *m *distinct districts (*m *= 8), and denote the observed number of malaria cases as *O*_*i *_with the corresponding expected number of cases *E*_*i *_Expected counts are calculated by multiplying the district population density by the country malaria mortality reference rate (WHO and UNICEF) chosen from the reference population, i.e. *E*_*i *_= *P*_i _* *R *where *P*_*i *_and *R *are district population density and mortality reference rate respectively. Reference population is chosen to be infants between 0 - 4 years of age because they lack parasite immunity thus being the most vulnerable portion of population. Conditional on *i *the counts *O*_*i *_of individual regions follow a Poisson distribution yielding stochastic variables with distribution *O*_*i *_~ *Pois*(*E*_*i*_*μ*_*i*_) (1), where *μ*_*i *_is the area specific risk rate.

The relative risk parameter *μ*_*i *_is assigned a log-normal prior distribution where the mean and variance are defined as a linear function of common intercept term *α *and two independent random effects. The term *u*_*i *_represents the correlated heterogeneity reflecting local spatial structure by incorporating the influence of neighbouring geographic areas, and *v*_*i *_is the uncorrelated heterogeneity that does not depend on geographic location (exchangeable). Prior distributions are assigned to these linear terms and hyper-prior distributions assigned to variance terms, creating additional 3-levels of the hierarchy where the first is (1) above, as follows:

Level 2: log *μ*_*i *_= *α *+ *u*_*i *_+*v*_*i *_(2).

The inclusion of environmental covariates temperature and rainfall into (2) yields the model, , where *i *= 1,..., *m *(2.1).

*X*_*ki *_= (*x*_*k*1_,..., *x*_*km*_) - is the ecological vector of covariates, and *β *is a vector of regression coefficients *β *= (*β*_1_, *β*_2_), with *β*_1 _the maximal temperature and *β*_2 _the rainfall quantity coefficients respectively. This model belongs to a class of models generally known as *convolution regression models*.

Level 3: Intercept term *α *is assigned a flat prior and non-informative prior distribution *β*_*k *_~ *N*(0.0,1.0*E *- 05) is assigned to regression coefficients with high variance. Excess heterogeneity *v*_*i *_is modelled through a set of exchangeable priors with zero mean and variance  given by,  (3).

A Conditional Autoregressive CAR (1) model proposed by [**15**] is used, where  (4). The term  is the weighted mean of the distribution, and  the variance. The contiguity values *w*_*ij *_= 1 for districts *i and j *that are adjacent (i.e. neighbours), and *w*_*ij *_= 0 otherwise. Parameters  and  are used to control the variability of spatial effects. Inverse Gamma prior distribution specified to all the variance parameters, with shape *a *= 0.5 and scale *b *= 0.0005.

The model was fitted in Winbugs [16], with Geobugs used for mapping the resulting posterior distribution of the RR estimated parameters. To speed up convergence, the model was re-parameterized by centring the covariates on their mean and divided by the corresponding standard deviation. Two parallel chains were run with over-dispersed starting values that were a combination of posterior means from a trial run for each year [17]. A burn-in of 1,000 interactions followed by 200,000 interactions was allowed and the values of main parameters were stored. Convergence of stored variables was checked through the analysis of the Brooks, Gelman and Rubin statistics [18], and by visual examination of history and density plots. The level of autocorrelation in chains was verified by using the built-in autocorrelation function of Winbugs. The intercept and covariate coefficients were still auto-correlated with first lag values around 0.75-0.90. To reduce the level of autocorrelation thinning was performed. A further 100 000 interactions were then run to collect the posterior distribution of each parameter. This number of interactions had been determined to fully describe the posterior distributions as the ratio Monte Carlo error and standard deviation was found to be less than 0.05 for all parameters. DIC statistic was calculated for the following Bayesian models (where random effects were included):

• With and without covariates;

• With either one of covariates.

This led to determining whether the fit of the model was affected by addition or suppression of covariates.

## Results

The inference is based on a sample of around 900,000 malaria cases observed in two years. Table 1 illustrates the overall mean relative risk (i.e. the baseline malaria intercept), the median malaria distribution and also the lower and upper posterior interval limits in the fully model version (all covariates).

**Table 1:** Baseline malaria intercept, median and 95%CI for year and season in Maputo province

Year and season	Mean	Lower limit (2.50%)	Median	Upper limit (97.50%)
*2001 - Rainy/Summ*er	0.2286	0.009099	0.1516	0.6998

2001 - Dry/Winter	0.7513	9.63E-04	0.2329	5.646

2002 - Rainy/Summer	0.03168	6.83-E-04	0.0143	0.1307

*2002 - Dry/Winter*	0.7903	0.08158	0.487	3.028

Posterior estimates and credibility intervals of the parameters are presented in table 2 and table 3. In summer season of both years, the rainfall covariate is not associated with malaria incidence in the model where it is considered the only covariate. While temperature is associated to malaria incidence in all the models it is included. In the dry season of year 2001, the temperature is not associated to malaria incidence in the model with both covariates. However, it is associated to malaria in the model with temperature covariate. Rainfall is associated to malaria incidence in the models with both covariates and with rain only. For year 2002, temperature is not associated with malaria incidence in the model of temperature covariate and the rainfall is not associated in model with both covariates. The rainfall environmental factor however, is associated with malaria in the model with rainfall covariate, while temperature is associated in the model with both covariates.

**Table 2:** Posterior estimates of regression coefficients and variances of random effects of different models for winter

Model	Alpha	**Beta**[1]	**Beta**[2]	Variance U	Variance V	DIC
**Year 2001**						
**No covariates**	1.11 (0.79, 1.43)	-	-	0.0035 (2.1E-04, 1.37)	0.19 (7.6E-04,0.699)	106.9
**Temperature covariate**	3.53 (1.22,6.12)	3.54 (0.27, 7.38)	-	0.0038 (2.14E-04, 1.4)	0.191 (7.6E-04, 0.69)	106.9
**Rainfall covariate**	3.8 (1.32, 6.27)	-	4.12 (0.32, 7.95)	0.0034 (2.1E-04, 1.34)	0.195 (8.7E-04, 0.71)	106.9
**Both covariates**	-1.46 (-6.95,1.73)	-8.88 (-18.99,-3.33)	2.25 (-2.58,17.7)	0.0044 (2.1E- 04,1.51)	0.183 (5.93E-04,0.69)	106.9
**Year 2002**						
**No covariates**	1.33 (1.02, 1.61)	-	-	0.0089 (2.3E-04,1.72)	0.18 (4.2E-04,0.72)	106.8
**Temperature covariate**	2.22 (-1.75,5.67)	-2.42 (-16.3, 11.7)	-	0.009 (2.2E-04, 1.75)	0.185 (4.0E-04, 0.77)	106.9
**Rainfall Covariate**	3.32 (-0.79, 7.11)	-	4.11 (-4.27,11.6)	0.078 (2.5E-04, 2.1)	0.133 (3.0E-04, 0.78)	106.9
**Both covariates**	-0.719 (-2.51, 1.11)	5.498 (0.696,12.2)	-0.223 (-6.26,4.06)	0.282 (2.7E-04, 2.04)	0.089 (2.9E-04,0.638)	106.8

Beta [1] - temperature and Beta [2] - rainfall. Credibility intervals at 95% in brackets

**Table 3:** Posterior estimates of regression coefficients and variances of random effects of different models for summer

Model	Alpha	**Beta**[1]	**Beta**[2]	Variance U	Variance V	DIC
**Year 2001**						
**No covariates**	1.12 (0.82, 1.47)	-	-	0.0042 (2.2E-04, 1.62)	0.18 (5.4E-04,0.67)	112.5
**Temperature covariate**	1.12 (-2.16, 3.81)	0.014 (-3.94,4.69)	-	0.0042 (2.1E-04, 1.48)	0.182 (6.1E-04, 0.67)	112.3
**Rainfall covariate**	1.62 (-1.15, 5.36)	-	-0.851 (-6.54,3.28)	0.0048 (2.2E-04, 1.54)	0.18 (5.57E-04,0.67)	112.5
**Both covariates**	-1.89 (-4.7, -0.36)	1.99 (0.56,4.13)	2.83 (0.39, 5.04)	0.0036 (2.1E-04, 1.35)	0.192 (8.83E-04,0.69)	112.4
**Year 2002**						
**No covariates**	1.34 (1.11, 1.65)	-	-	0.0052 (2.2E-04, 1.49)	0.196 (5.6E-04,0.72)	112.3
**Temperature covariate**	-2.23 (-5.22,0.99)	1.62 (0.18, 2.97)	-	0.027 (2.48E-04,1.78)	0.16 (3.58E-04,0.68)	112.4
**Rainfall Covariate**	1.73 (-1.27, 4.58)	-	-0.999 (-10.6, 8.8)	0.0072 (2.3E-04,1.73)	0.184 (3.71E-04,0.71)	112.4
**Both covariates**	-4.25 (-7.29,-2.04)	2.23 (1.46, 2.85)	4.71 (-3.54,11.3)	0.034 (2.4E-04,1.93)	0.143 (3.4E-04,0.679)	112.4

Beta [1] - temperature and Beta [2] - rainfall. Credibility intervals at 95% in brackets

The results of model comparison for summer 2001 showed that the spatial model with temperature only had relatively small DIC value, being the model which best fit the data. In winter 2001, all models had the same DIC value. In this case the simplest model, i.e. the spatial model with no covariates is chosen. The estimates and CI 95% of random effects in all the models are very similar.

In winter 2002 the results of model comparison showed that models with no covariate and with both covariates had small DIC value. The variation of malaria incidence due to structured random effect obtained in the spatial model with both covariates is higher compared to model with no covariate. While for the unstructured random effect the estimates and CI 95% are very similar. Model comparison for summer 2002 showed that the spatial model with no covariates is the best-fit model as it had smaller DIC value of all. However, the spatial variation of unstructured random effect in model with no covariates (0.196, CI 95%: [5.6E-04, 0.72]) is very similar to the variance of the model with both covariates (0.143, CI 95%: [3.4E-04, 0.679]). To illustrate the mapped spatial malaria incidence risk and variation of random effects, see additional files:

• Additional file 1: Year 2001- both seasons

• Additional file 2: Winter 2002

• Additional file 3: Summer 2002

The results of spatial pattern of malaria incidence risk in 2001 show no seasonal variation of RR, where the highest incidence rate is from 4.6 to 5.9 cases per 1,000 population year in north districts of Magude and Moamba. Low incidence rate ranges from 1.5 to 1.9 in Matola in the centre and Matutuine in the south. For year 2002 the geographic pattern of RR in both seasons is over 1.9 cases per 1,000 population year with the highest value of 9.5 in district of Matola.

## Discussion

The objective of this investigation was to determine the association of malaria incidence with environmental variables temperature and rainfall in Maputo province, and mapping this variation. The study used yearly aggregated malaria data by district level and additional climate data sources to access the role of climate. Malaria incidence occurs in distinct seasons and the level of epidemics varies alternating from low to high incidence periods. To capture this pattern an assumption about malaria data distribution for each year was made. This assumption has yielded two sub-models per year, following climate seasonality of Mozambique: winter (dry) season in months of April-September and summer (wet) season in months October-March.

The results of the analysis confirm that the malaria cases in Maputo province were significantly associated with climate variables. The climate seasonal variation influences malaria incidence but does not significantly modify its spatial patterns.

Results from summer 2001 show that malaria incidence was strongly and positively correlated with temperature. For year 2002, malaria incidence was found to be associated with both rainfall and temperature. Similar results were found for winter 2002 season, with temperature strongly correlated to malaria incidence. Although the analysis has revealed association of malaria to rainfall only in year 2002 (summer), this may be misleading as there could be some undetermined relationships such as increase in stable mosquitoes breeding sites under low or non-existent rainfall. The average value of 26°C of maximum temperature in year 2001 may have accelerated the parasite development. Moreover, the study in [19] found that the most significant variables to malaria transmission in KwaZulu-Natal, South Africa were mean maximum daily temperature of the preceding months and rainfall of corresponding summer season. They suggest that the survival rate of mosquitoes or the size of parasite's reservoir could be determined by the temperature in the preceding rainy season. This would consequently make an increase in malaria incidence more likely on the following rains onset. The results obtained in this study agree with [7] regarding positive association of malaria incidence with rainfall, without adjusting however for the covariate maximum temperature.

Covariates temperature and rainfall do not explain all the variability present in the malaria data as there is overdispersion that is captured by regional structured and unstructured random effects. This can be seen on variation of structured and unstructured effects with values (0.0036 CI 95%: [2.1E-04, 1.35]) and (0.19 CI 95%: [8.83-E-04, 0.69]) in summer 2001, and also in winter 2002 with results (0.282 CI 95%: [2.6E-04, 2.04]) and (0.089 CI 95% [2.9E-04, 0.638]) respectively.

Produced maps illustrate spatial variation of malaria incidence, being different from climatic suitability model maps [15]. Their objective is to represent an empirical description of Malaria RR in Maputo province. They identify high malaria RR in districts of Matola over 6.9, Moamba over 5.9 and Magude over 4.9 in 1000 population year. Areas with medium RR are Boane and Manhiça with value over 2.9. Districts found to exhibit low malaria risk are Marracuene, Matutuine and Namaacha.

The analysis performed is expected to be of help to malaria control programmes on:

1. Identifying the areas where intervention may be required and perhaps which will be most appropriate (house-spraying, ITN usage, etc.)

2. Encouragement and improvement of malaria testing procedure/diagnostic of the disease. This may improve a systematic data collection and notification of malaria cases, reducing overestimation specially from clinically diagnosed cases

3. Improvement of decision-making process specially through the increase of feedback information to district level which will also encourage improved reporting

4. Awakening to the need for malaria environmental control by fighting the mosquito larvae

5. If needed, adjust treatment with most appropriate and modern drugs

6. Designing and implementation of emergency responses

Due to unavailability of surveillance malaria system in Mozambique, climate forecasts become important as it offers an acceptable degree of predictability of climate fluctuations at a seasonal lead time. Studies like this have great potential as they may help proof this hypothesis.

## Conclusions

Using yearly collected data from different health centres in each district, the linkage between malaria incidence and environmental data was investigated in Maputo province. The model made it possible to analyse the way malaria data cases arose under climate conditions that occurred, highlighting their crucial relationship.

Malaria incidence in Maputo province does not present an independent spatial pattern in relation to the seasonal climatic conditions in years 2001 and 2002. The change in any of the climatic variable has lead to a corresponding modification of incidence of malaria and its spatial pattern in the region. These findings may be useful for the planning of malaria control activities, as they may induce the design and implementation of more reliable malaria policy and intervention in the region.

Estimation of explanatory coefficients of the model indicated that temperature had a strong impact on malaria incidence in the region.

Although the climatic factors were assumed constant over the whole area and for each season, the study reveals the importance of spatial analysis in the research of interactions of tropical disease as malaria and the two environmental factors, rainfall and temperature in Maputo province. Furthermore, the investigation of associations of malaria incidence risk and environmental climatic factors in Maputo province and Mozambique is very important as it may bring more knowledge on the epidemiology distribution map of malaria. However, there remain some unmeasured factors that might relate to malaria incidence in Maputo province (type of housing, proximity to water bodies, etc.), which were captured in the model by structured and unstructured effects.

Studies that look at the relationship of malaria incidence and environmental variables in Maputo province are significant step towards the development of local surveillance malaria systems. They should be extended to the analysis of large malaria, climate and other datasets, and also include a temporal dimension.

## Abbreviations

MCMC: Markov Chain Monte Carlo; DIC: Deviance Information Criteria; NMCP: National Malaria Control Program; INAM: Mozambique National Meteorology Institute; RR: Relative risk; ITN: Insecticide-Treated bed Net; WHO: World Health Organization; UNICEF: United Nations International Children's Emergency Funds; INE: National Institute of Statistics; DINAGECA: National Directorate of Geography and Cadastre; UEM: Universidade Eduardo Mondlane; SIDA: Swedish International Development Cooperation Agency; CI: Credibility Interval; BES: Weekly Epidemiological Bulletin.

## Competing interests

The authors declare that they have no competing interests.

## Authors' contributions

MA was responsible for analyses and interpretation of results and manuscript's revision. OPZ conceived and designed the study, data cleaning, performed the analysis and results interpretation, and manuscript preparation. The authors read and approved the manuscript.

## Supplementary Material

Additional file 1 Contains 2001 maps of RR, structured and unstructured random effects for both seasons. Click here for file

Additional file 2 Contains 2002 maps of RR, structured and unstructured random effects winter season. Click here for file

Additional file 3 Contains 2002 maps of RR, structured and unstructured random effects for summer season. Click here for file
