# Supplementary material for: Mapping malaria incidence distribution that accounts for environmental factors in Maputo Province - Mozambique
Source: Malar J. 2010 Mar 21;9:79. doi: 10.1186/1475-2875-9-79 (PMC2853555; doi:10.1186/1475-2875-9-79)
Supplement: Additional file 1 — Contains 2001 maps of RR, structured and unstructured random effects for both seasons. [file 1475-2875-9-79-S1.PDF]

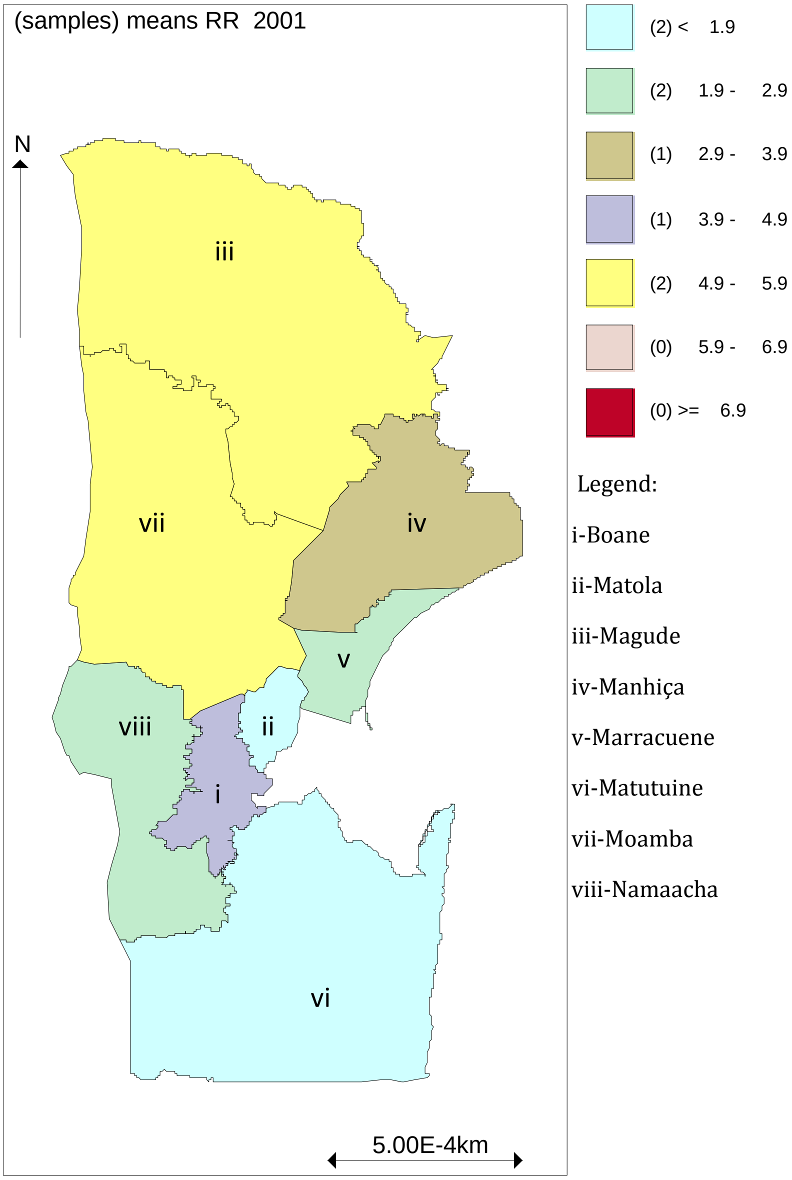

Note: RR maps of winter and summer in year 2001 are similar. Same applies to structured maps of both seasons. Thus, the same map is shown for each case and season respectively.

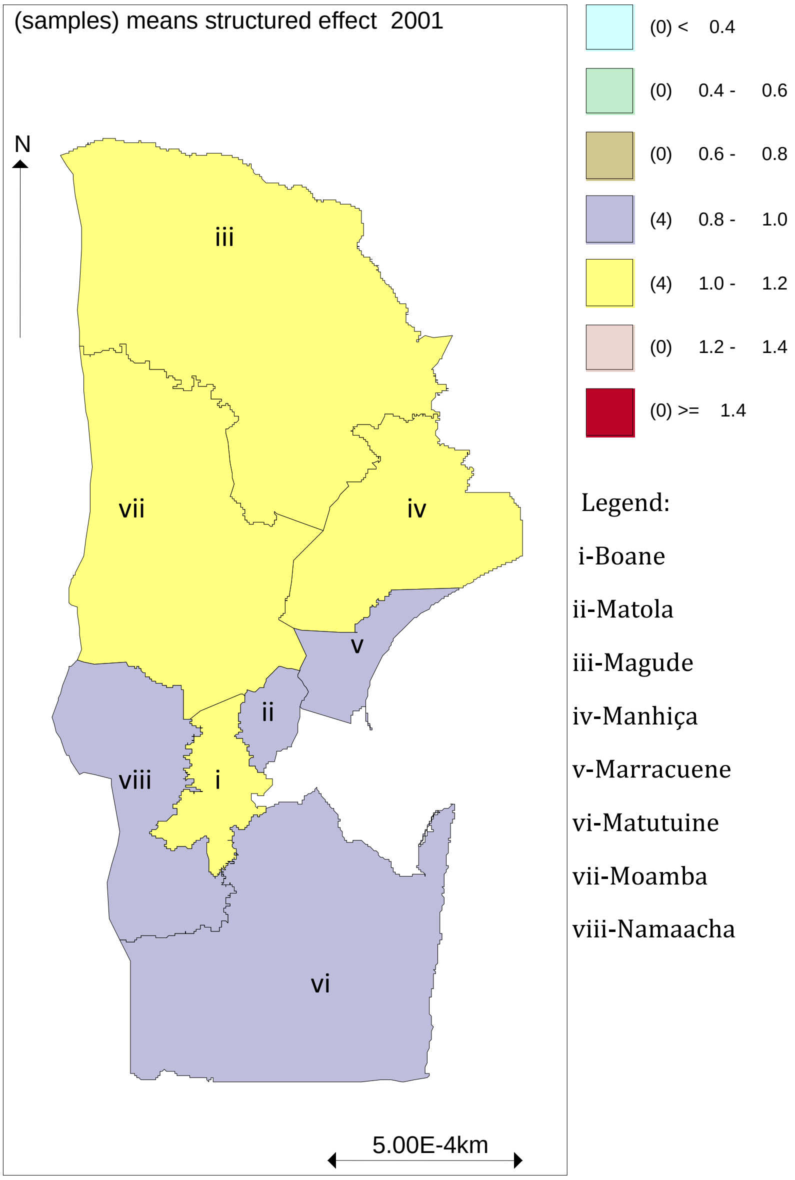
(samples) means structured effect 2001
N
iii
vii
iv
v
ii
viii
i
vi
5.00E-4km
(0) < 0.4
(0) 0.4 - 0.6
(0) 0.6 - 0.8
(4) 0.8 - 1.0
(4) 1.0 - 1.2
(0) 1.2 - 1.4
(0) >= 1.4
Legend:
i-Boane
ii-Matola
iii-Magude
iv-Manhiça
v-Marracuene
vi-Matutuine
vii-Moamba
viii-Namaacha

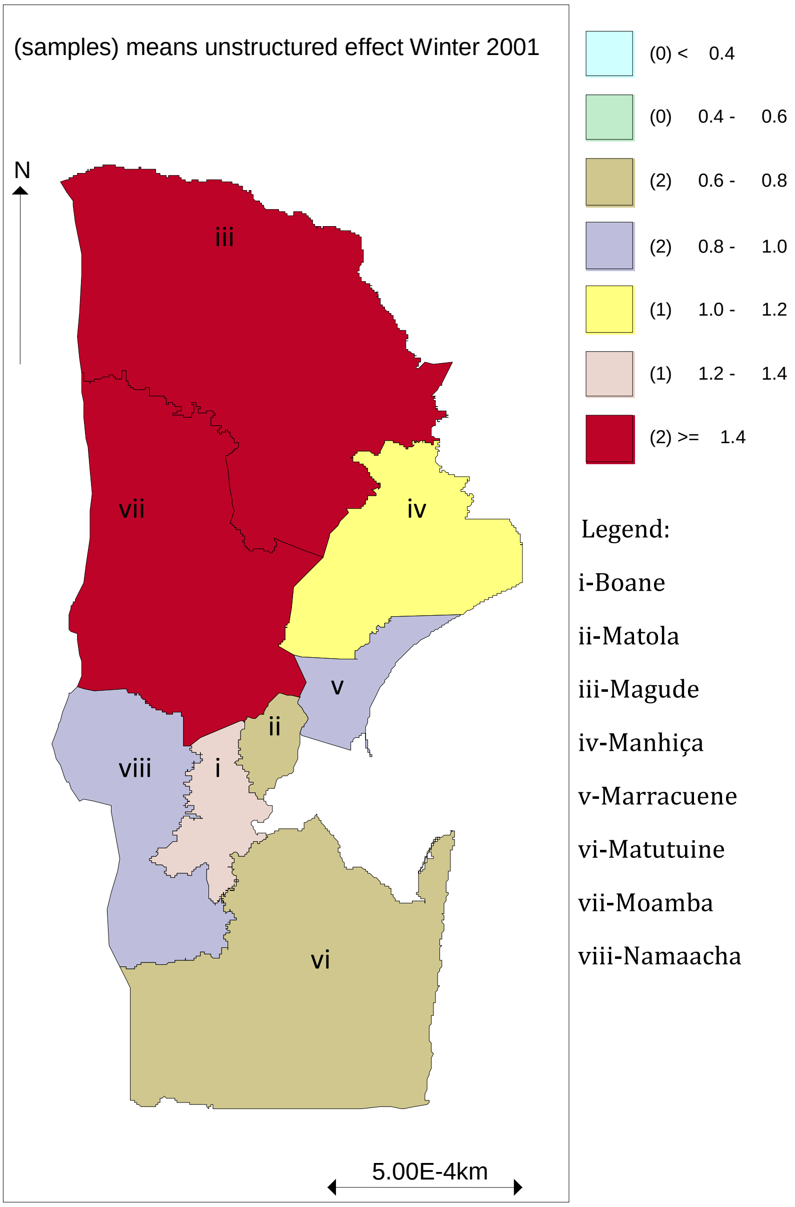
(samples) means unstructured effect Winter 2001
N
iii
vii
iv
v
ii
viii
i
vi
5.00E-4km
(0) < 0.4
(0) 0.4 - 0.6
(2) 0.6 - 0.8
(2) 0.8 - 1.0
(1) 1.0 - 1.2
(1) 1.2 - 1.4
(2) >= 1.4
Legend:
i-Boane
ii-Matola
iii-Magude
iv-Manhiça
v-Marracuene
vi-Matutuine
vii-Moamba
viii-Namaacha

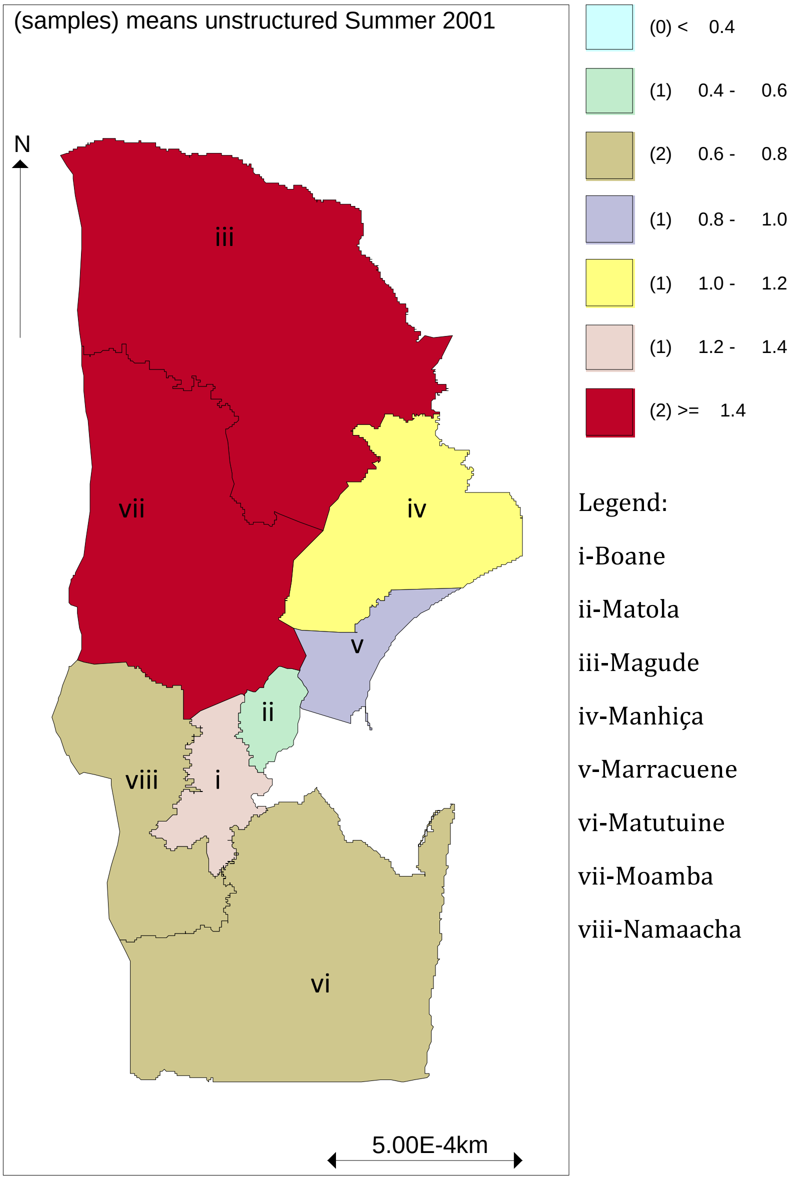
(samples) means unstructured Summer 2001
N
iii
vii
iv
v
ii
viii
i
vi
5.00E-4km
(0) < 0.4
(1) 0.4 - 0.6
(2) 0.6 - 0.8
(1) 0.8 - 1.0
(1) 1.0 - 1.2
(1) 1.2 - 1.4
(2) >= 1.4
Legend:
i-Boane
ii-Matola
iii-Magude
iv-Manhiça
v-Marracuene
vi-Matutuine
vii-Moamba
viii-Namaacha
