# Supplementary material for: Mapping malaria incidence distribution that accounts for environmental factors in Maputo Province - Mozambique
Source: Malar J. 2010 Mar 21;9:79. doi: 10.1186/1475-2875-9-79 (PMC2853555; doi:10.1186/1475-2875-9-79)
Supplement: Additional file 3 — Contains 2002 maps of RR, structured and unstructured random effects for summer season. [file 1475-2875-9-79-S3.PDF]

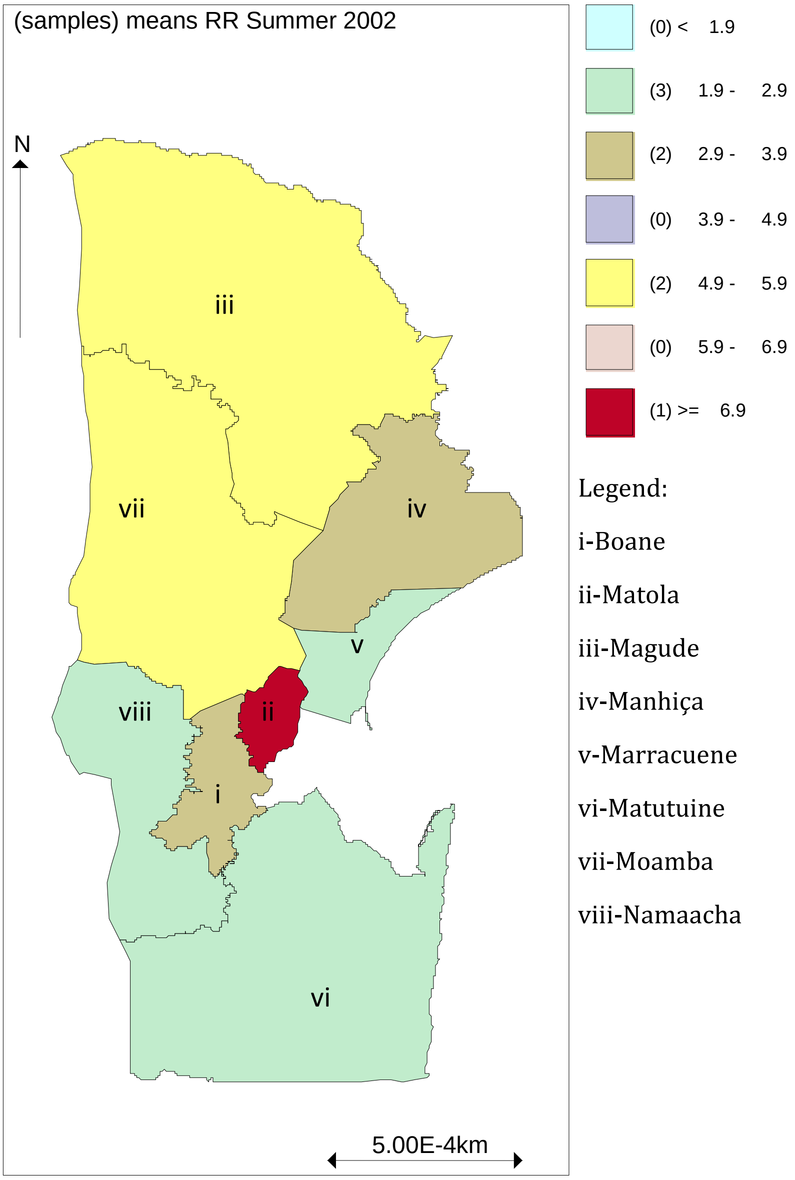

Legend:

i-Boane

ii-Matola

iii-Magude

iv-Manhiça

v-Marracuene

vi-Matutuine

vii-Moamba

viii-Namaacha

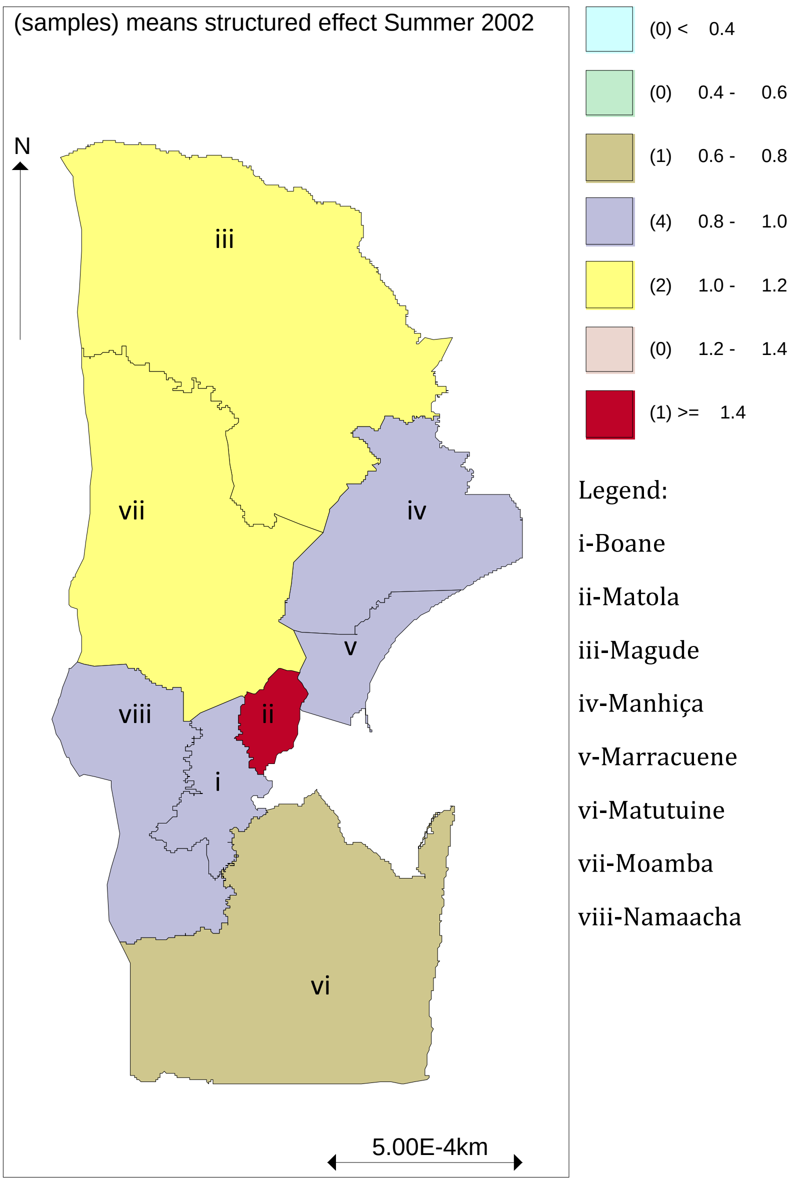
(samples) means structured effect Summer 2002
N
iii
vii
iv
v
viii
ii
i
vi
5.00E-4km
(0) < 0.4
(0) 0.4 - 0.6
(1) 0.6 - 0.8
(4) 0.8 - 1.0
(2) 1.0 - 1.2
(0) 1.2 - 1.4
(1) >= 1.4
Legend:
i-Boane
ii-Matola
iii-Magude
iv-Manhiça
v-Marracuene
vi-Matutuine
vii-Moamba
viii-Namaacha

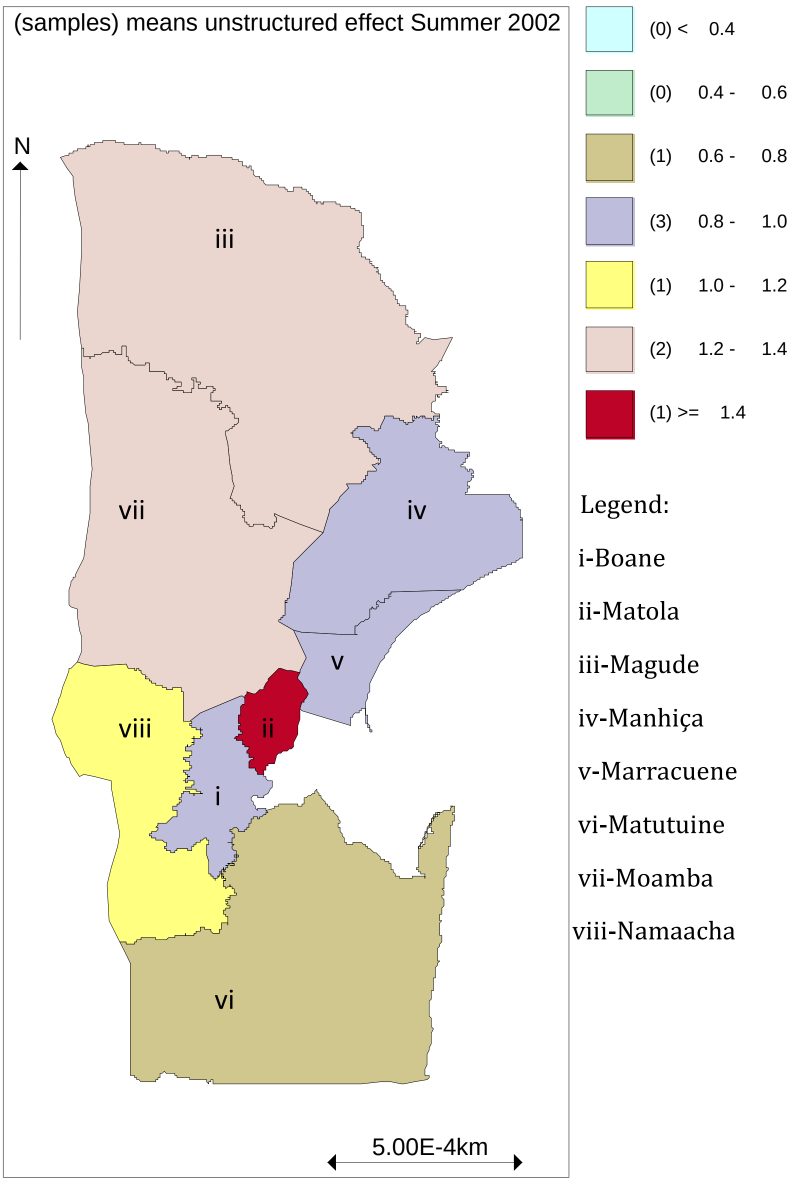
(samples) means unstructured effect Summer 2002
N
iii
vii
iv
v
viii
ii
i
vi
5.00E-4km
(0) < 0.4
(0) 0.4 - 0.6
(1) 0.6 - 0.8
(3) 0.8 - 1.0
(1) 1.0 - 1.2
(2) 1.2 - 1.4
(1) >= 1.4
Legend:
i-Boane
ii-Matola
iii-Magude
iv-Manhiça
v-Marracuene
vi-Matutuine
vii-Moamba
viii-Namaacha
